# A randomized, double-blind phase 2b trial to evaluate efficacy of ChAd63-KH for treatment of post kala-azar dermal leishmaniasis

**DOI:** 10.1016/j.omtm.2024.101310

**Published:** 2024-07-30

**Authors:** Brima M. Younis, Rebecca Wiggins, Eltahir A.G. Khalil, Mohamed Osman, Francesco Santoro, Chiara Sonnati, Ada Keding, Maria Novedrati, Giorgio Montesi, Ali Noureldein, Elmukashfi T.A. Elmukashfi, Ala Eldin Mustafa, Mohammed Alamin, Mohammed Saeed, Khalid Salman, Ahmed J. Suliman, Amin E.A. Musa, Alison M. Layton, Charles J.N. Lacey, Paul M. Kaye, Ahmed M. Musa

**Affiliations:** 1 Department of Clinical Pathology & Immunology, Institute of Endemic Diseases, University of Khartoum, Khartoum 11111, Sudan; 2 York Biomedical Research Institute, Hull York Medical School, University of York, Heslington, York YO10 5DD, UK; 3 Department of Medical Biotechnologies, University of Siena, 53100 Siena, Italy; 4 Department of Health Sciences, University of York, Heslington, York YO10 5DD, UK

**Keywords:** *Leishmania*, post kala-azar dermal leishmaniasis, vaccine, therapeutic, adenovirus, clinical trial

## Abstract

In a recent phase 2a clinical trial, the candidate leishmaniasis vaccine ChAd63-KH was shown to be safe and immunogenic in Sudanese patients with post kala-azar dermal leishmaniasis (PKDL). However, its value as a stand-alone therapeutic was unknown. To assess the therapeutic efficacy of ChAd63-KH, we conducted a randomized, double-blind, placebo-controlled phase 2b trial (ClinicalTrials.gov: NCT03969134). Primary outcomes were safety and efficacy (≥90% improvement in clinical disease). Secondary outcomes were change in severity grade and vaccine-induced immune response. 86 participants with uncomplicated PKDL of ≥6 month duration were randomized to receive ChAd63-KH (7.5 × 10^10^ viral particles, once by the intramuscular route) or placebo. 75 participants (87%) completed the trial as per protocol. No severe or serious adverse events were observed. At day 90 post-vaccination, 6/40 (15%) and 4/35 (11%) participants in the vaccine and placebo groups, respectively, showed ≥90% clinical improvement (risk ratio [RR] 1.31 [95% confidence interval (CI), 0.40–4.28], *p* = 0.742). There were also no significant differences in PKDL severity grade between study arms. Whole-blood transcriptomic analysis identified transcriptional modules associated with interferon responses and monocyte and dendritic cell activation. Thus, a single vaccination with ChAd63-KH showed no therapeutic efficacy in this subset of Sudanese patients with PKDL.

## Graphical abstract

**Figure:**
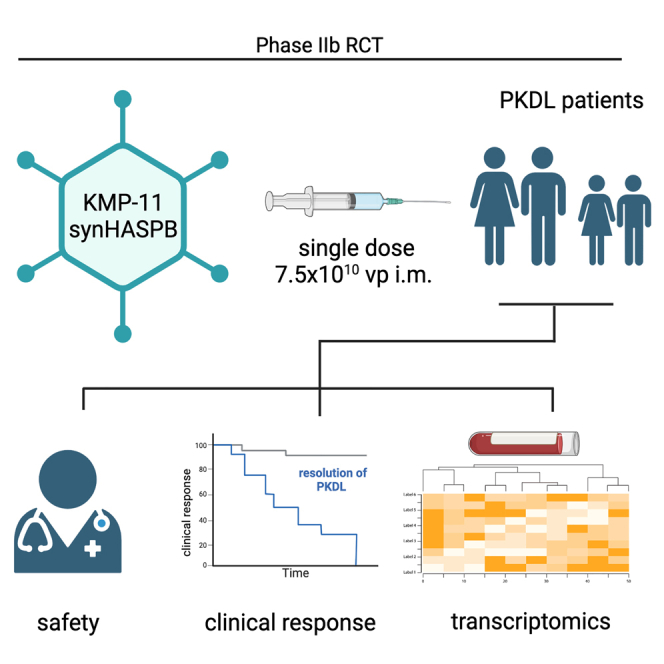

## Teaser

Post kala-azar dermal leishmaniasis (PKDL) is a poorly understood chronic skin disease with limited treatment options. Here, Younis and colleagues report that in a randomized, placebo-controlled phase 2b clinical trial, therapeutic vaccination with an adenovirus-based leishmaniasis vaccine did not provide therapeutic benefit in Sudanese patients with PKDL.

## Introduction

Post kala-azar dermal leishmaniasis (PKDL) is a chronic dermatological sequela associated with treatment for visceral leishmaniasis (VL; kala-azar), but it may occur without a history of VL or even during treatment of VL (when it is known as para kala-azar dermal leishmaniasis). PKDL typically involves the face and later spreads to the extremities and trunk.^1^^,^^2^^,^^3^ PKDL has often been confused with leprosy, and because of its chronic but not debilitating nature, it is often tolerated by infected people, who fail to seek treatment. Case rates for PKDL are intimately linked to the waxing and waning of VL incidence.^4^ The VL elimination campaign in South Asia has served to focus attention on PKDL, as those with the disease have been shown to harbor parasites in their skin and be infectious to the sand fly vector. As such, people with PKDL may serve to maintain infection in inter-epidemic periods, thus posing a threat to elimination.^5^ Epidemiological modeling has demonstrated the value of a PKDL vaccine for mitigating this risk in a post-elimination era.^6^ Recent calls to action for VL elimination on the African continent also recognize the need to have effective means to control PKDL.^7^ Drug regimens for PKDL are generally arduous and invasive and have multiple potential side effects.^8^^,^^9^ PKDL can also be exacerbated by HIV, and such cases respond poorly to treatment.^10^ New combination therapies have been evaluated and show promise both in reducing the burden of PKDL^11^ and in treatment,^12^ but there has been a strong and persistent argument for developing additional means of control, including therapeutic vaccines.^13^^,^^14^^,^^15^

PKDL has several intriguing features epidemiologically, clinically, and immunologically. VL is caused by infection with two species of the protozoan parasite *Leishmania*, namely *L. donovani* and *L. infantum*, yet PKDL is restricted to infection with *L. donovani* and found only in the Old World.^2^ PKDL is also restricted within the geographical range of *L. donovani*, being found in Sudan and South Asia but rarely in other countries in East Africa. PKDL typically occurs after treatment, whether this be with pentavalent antimonials, amphotericin B, miltefosine, or paromomycin/antimonial combinations.^16^ However, the onset of disease varies geographically, being rapid (weeks to months) in Sudan compared to delayed (often years) in South Asia. Clinical presentation also varies geographically and at different body sites, with various hypotheses, including ultraviolet light (UVB) exposure, being put forward to explain differences in clinical presentation.^17^^,^^18^ In Sudan, disease presents mainly as nodules and papules that, in ∼80% of cases, self-resolve over several months.^8^^,^^19^ In the remainder, lesions may persist, often for years. In contrast, in South Asia, cases can show hypopigmented macular lesions or be polymorphic, with both macular and nodular lesions. Immunological and histopathological differences have also been noted.^17^^,^^18^^,^^20^^,^^21^^,^^22^^,^^23^ Collectively, these features suggest that PKDL is a heterogeneous disease, with implications for the development of new therapeutics.

Therapeutic vaccination against PKDL in Sudan has its historical roots in the use of first-generation vaccines for VL. Khalil and colleagues conducted phase 1/2 randomized trials in volunteers with no history of VL and showed that a two intradermal doses of autoclaved *L. major* (ALM) administered with Bacille Calmette-Guérin (BCG) was safe, well tolerated, and immunogenic, as measured by skin test conversion.^24^^,^^25^^,^^26^ However, no efficacy with regards to protection against VL was observed. A follow-up study of alum-adjuvanted ALM + BCG also demonstrated safety and immunogenicity,^27^^,^^28^ and this formulation was evaluated in combination with sodium stibogluconate (SSG) as a potential therapeutic in patients with PKDL with persistent disease, reporting a cure rate at 60 days of 87% in the combined therapy group vs. 53% in the SSG alone group (SSG + vaccine efficacy = 71%, 95% confidence interval [CI] for risk ratio [RR], 0.7–1.16).^29^ Based on these encouraging results, we first conducted an open-label phase 2a trial (LEISH2a; ClinicalTrials.gov: NCT02894008) in patients with persistent PKDL of ChAd63-KH, a new adenoviral vaccine incorporating two well-documented candidate antigens.^30^ This study confirmed that ChAd63-KH was both safe and immunogenic in this patient group,^31^ opening the way for the randomized, controlled efficacy trial reported here.

## Results

### Site initiation and recruitment

**Figure 1:**
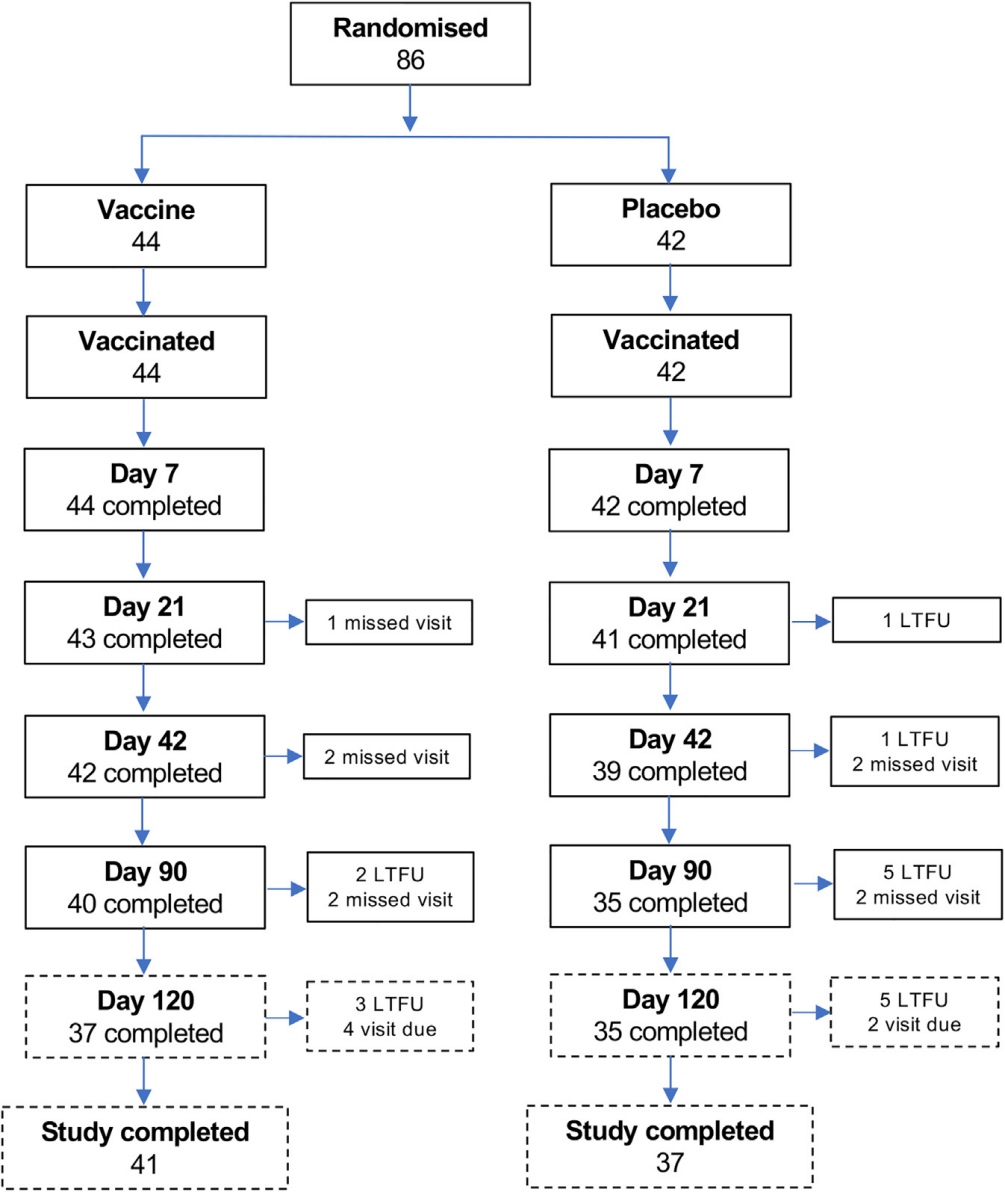
Study CONSORT diagram Solid boxes indicate participants to primary outcome at day 90 post-vaccination (Vx). Treatment was offered per protocol from day 90 (see main text). Dotted boxes were outside the trial window for primary outcome and represent scheduled follow-up to provide or monitor standard of care. LTFU, lost to follow up

The LEISH2b study was conducted over approximately 4 years, punctuated by periods of significant challenge that resulted in temporary trial suspensions. The site initiation visit, attended by all field site personnel, trial monitors, and trial staff from the Universities of Khartoum and York, took place in August 2019 in Addis Ababa due to a popular uprising and deteriorating security situation in Sudan. Recruitment began on April 4, 2020, and continued through June 2020, with 17 participants recruited in this period. Recruitment was then paused due to the severe acute respiratory syndrome coronavirus 2 (SARS-CoV-2) pandemic. The second round of recruitment took place between December 2020 and February 2021, enrolling 28 participants. A third round of recruitment took place in April and May 2021, adding 23 participants. Delayed by a military coup, the final round of recruitment (May to June 2022) added 18 more, bringing the total to 86 participants. Following discussion with the LEISH2b data safety and monitoring board (DSMB), a meeting of the trial steering group with the funders was convened. All attendees agreed that recruitment should halt at 86 participants due to logistical difficulties in completing the trial prior to expiry of the investigational medicinal product (IMP) and the onset of further military clashes. 75/86 (87%) of enrolled participants completed the study to day 90 post-vaccination, with the last patient last visit on September 20, 2022. Missed visits and losses to follow-up were due to occupational priorities. A further day 120 visit was arranged to allow follow-up of any drug treatment provided at the completion of the study. A summary of study recruitment is provided in the CONSORT diagram (Figure 1).

### Study population

Demographics and PKDL grading of the 86 participants enrolled in this study are shown in Table 1. All participants had PKDL for a 6 month duration or longer, with the majority (88%) graded as either PKDL grade 1 or 2 (Tables 1 and S1). Day 90 visits were completed by 75 (87%) participants (*n* = 11 missed this time point or had been lost to follow-up; Figure 1). As per protocol, participants were offered standard of care (AmBisome) at day 90 if clinical improvement was less than 90%. Overall, 26/86 (30%) elected to receive treatment (8/20 adults; 18/66 adolescents), and these participants remain in long-term follow-up to confirm drug effectiveness.

**Table 1:** Baseline characteristics of participants in LEISH2b trial

Characteristics	Vaccine, *N* = 44	Placebo, *N* = 42
**Adults**	*n* = 11	*n* = 9
Sex		
Male	*n* = 8 (73%)	*n* = 6 (67%)
Female	*n* = 3 (27%)	*n* = 3 (33%)
PKDL grade		
1	*n* =7 (64%)	*n* =2 (22%)
2	*n* =3 (27%)	*n* =5 (56%)
3	*n* =1 (9%)	*n* =2 (22%)
Age (years)		
Mean (SD)	23.2 (5.98)	24.3 (7.18)
Median	21	22
Min max	18, 37	18, 40
BMI		
Mean (SD)	19.8 (1.92)	20.4 (1.67)
Median	20.1	19.6
Min max	16.2, 23.1	18.8, 23.3
**Adolescents**	*n* = 33	*n* = 33
Sex		
Male	*n* = 17 (52%)	*n* = 15 (45%)
Female	*n* = 16 (48%)	*n* = 18 (55%)
PKDL grade		
1	*n* = 14 (42%)	*n* = 18 (55%)
2	*n* = 15 (45%)	*n* = 12 (36%)
3	*n* = 4 (12%)	*n* = 3 (9%)
Age (years)		
Mean (SD)	13.4 (1.78)	13.0 (1.49)
Median	12	12
Min max	12, 17	12, 17
BMI		
Mean (SD)	17.2 (2.85)	16.2 (2.01)
Median	16.9	15.9
Min max	12.4, 26.9	12.9, 20.7

### Safety outcomes

**Figure 2:**
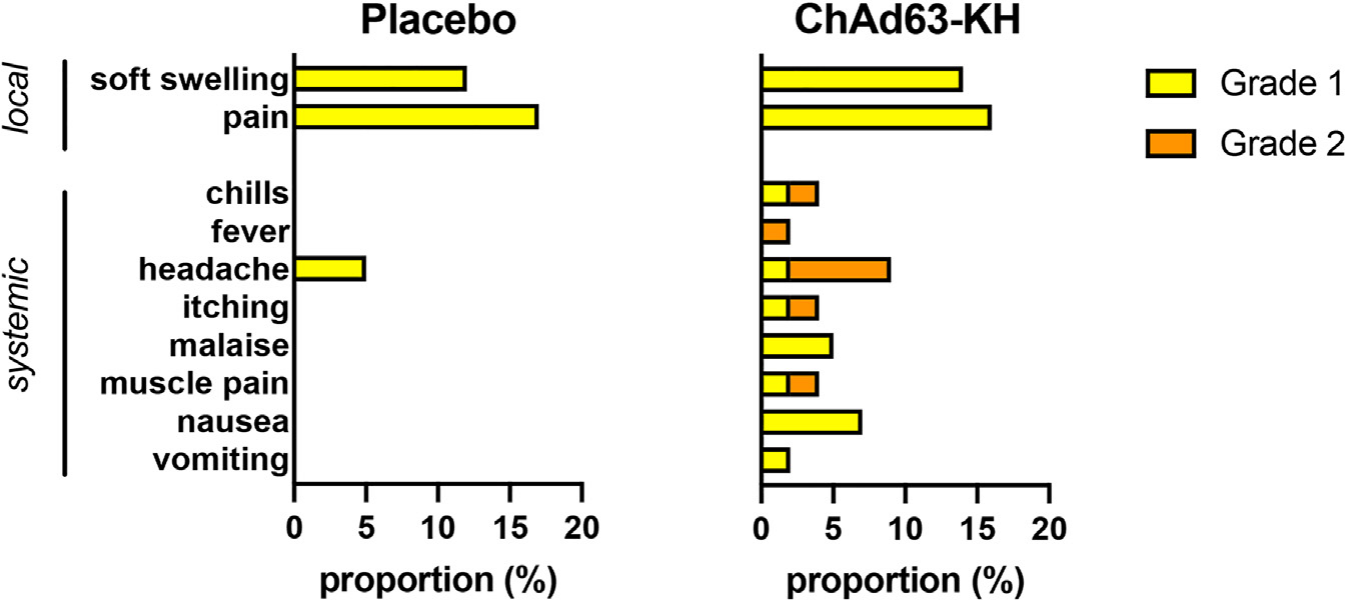
Adverse events associated with the LEISH2b trial Data are shown for local and systemic adverse events judged to be possibly, probably, likely, or definitely associated with Vx. Data are shown as proportions of participants in the study (*n* = 86). Only grade 1 AEs (mild, yellow; 26 vs. 17 events overall in vaccine vs. placebo groups) and grade 2 AEs (moderate; orange; 17 vs. 10 events overall in vaccine vs. placebo groups) were observed. See Tables S2–S5 for full details.

There were 70 (25 local and 45 systemic) adverse events (AEs) reported by 43 participants during the study, split 1.6:1 between the vaccine and placebo groups (Tables S2–S5). The average numbers of local AEs per participant were 0.30 in the vaccine vs. 0.29 in the placebo arms (median of 0 in both arms), and systemic AEs per participant were 0.68 in the vaccine vs. 0.36 in the placebo arms (median of 0 in both arms). Event numbers did not significantly differ between arms (Mann-Whitney U *p* = 0.921 and *p* = 0.501 for local and systemic events, respectively). AEs were limited to grade 1 and 2, with no grade 3 AEs, serious AEs (SAEs), or suspected unexpected serious adverse reactions (SUSARs) reported. All local and systemic AEs were deemed to be not serious and recovered. 44 AEs (25 local and 19 systemic) were considered possibly, probably, or definitely related to vaccination, again showing a bias toward vaccine recipients (Figure 2). These included itch, pain or soft swelling at the injection site, headache, vomiting, fever, and general muscle pain. Medication was not required for any of the local AEs, but paracetamol (for headache, chills, and fever) and chlorphenamine (for whole-body itch) were prescribed for systemic AEs. No clinically relevant changes in blood biochemistry or hematology were observed. The most common systemic AE unrelated to the study was malaria (16 clinical episodes, 15 patients). One case of thrombocytopenia was recorded but deemed unrelated to vaccination (occurring in the placebo group). Since the completion of the study, there have been no formal follow-up visits, but participants remain able to contact the study team should they have any future health issues. None have been reported to date.

### Clinical outcomes

**Figure 3:**
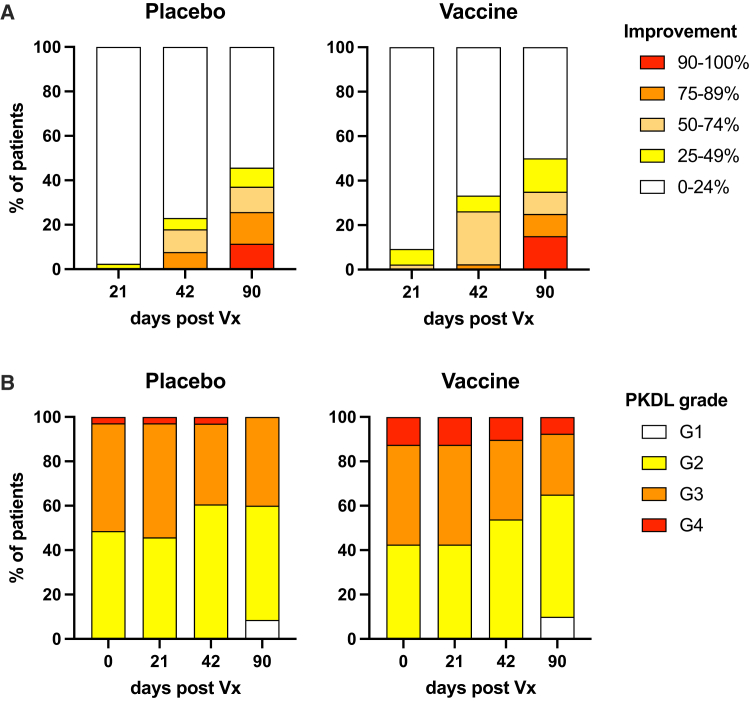
Clinical improvement in LEISH2b participants (A) Clinical improvement by study arm (*n* = 40 and *n* = 35 for vaccine and placebo groups, respectively). Participants were categorized based on degree of improvement at different times post-Vx. Color key indicates improvement category. No significant differences were detected at any time point or over time (Fisher’s exact test; see main text). (B) Distribution of overall PKDL grades by study arm (*n* = 40 and *n* = 35 for vaccine and placebo groups, respectively). Participants were scored for PKDL grade at Vx (day 0) and at indicated days post-Vx. Color key indicates grades. No significant differences were detected at any time point or over time (Fisher’s exact test; see main text).

The per-protocol primary outcome measure was 90% or greater improvement in clinical PKDL as determined by two clinical assessors. Of the 75 participants that completed the study to day 90 (39 female [F], 36 male [M]; Table S1), 6/40 (15%; 2 F, 4 M) and 4/35 (11%; 3 F, 1 M) in the vaccine and placebo arms, respectively, reached this threshold (RR 1.31 [95% CI, 0.40–4.28], *p* = 0.742). To explore whether there was any vaccine effect at lower levels of improvement, we calculated the proportion of participants that attained 25%, 50%, 75%, and 90% of improvement over time of follow-up, and the trajectories of recovery appeared to be very similar between arms (Figure 3A). Of note, all participants that reached the primary outcome did so between days 42 and 90 of follow-up. The secondary outcome of PKDL grading (grade 1 to grade 4) was evaluated, as PKDL severity is often used to monitor disease status.^32^ Grade distributions improved only marginally over time and were similar between arms (*p* = 0.36, *p* = 0.53, and *p* = 0.38 for days 21, 42, and 90, respectively; Fisher’s exact test; Figure 3B).

As duration of PKDL has been suggested to influence cure rate,^19^ we additionally obtained prior duration in months data for a subgroup of 57/75 participants completing day 90 follow-up (45 adolescents, 12 adults; 33 F, 24 M). We stratified this subgroup into two classes based on duration of PKDL (<18 months: median: 11 months, range: 6–11 months, *n* = 26; ≥18 months, median: 36 months, range: 18–120 months, *n* = 31) and evaluated whether there was any difference in the proportion of patients reaching a conservative 50% improvement. In the <18 month duration class, 6/11 receiving vaccine and 6/15 receiving placebo reached this threshold compared to 5/19 and 5/12 in the >18 month duration class. Hence, the duration of PKDL in this limited cohort did not appear to be associated with vaccine response or overall improvement.

Collectively, these analyses indicate that under the trial conditions employed, ChAd63-KH lacked efficacy as a single treatment in patients with persistent PKDL in Sudan.

### Whole-blood transcriptome prior to and after vaccination

**Figure 4:**
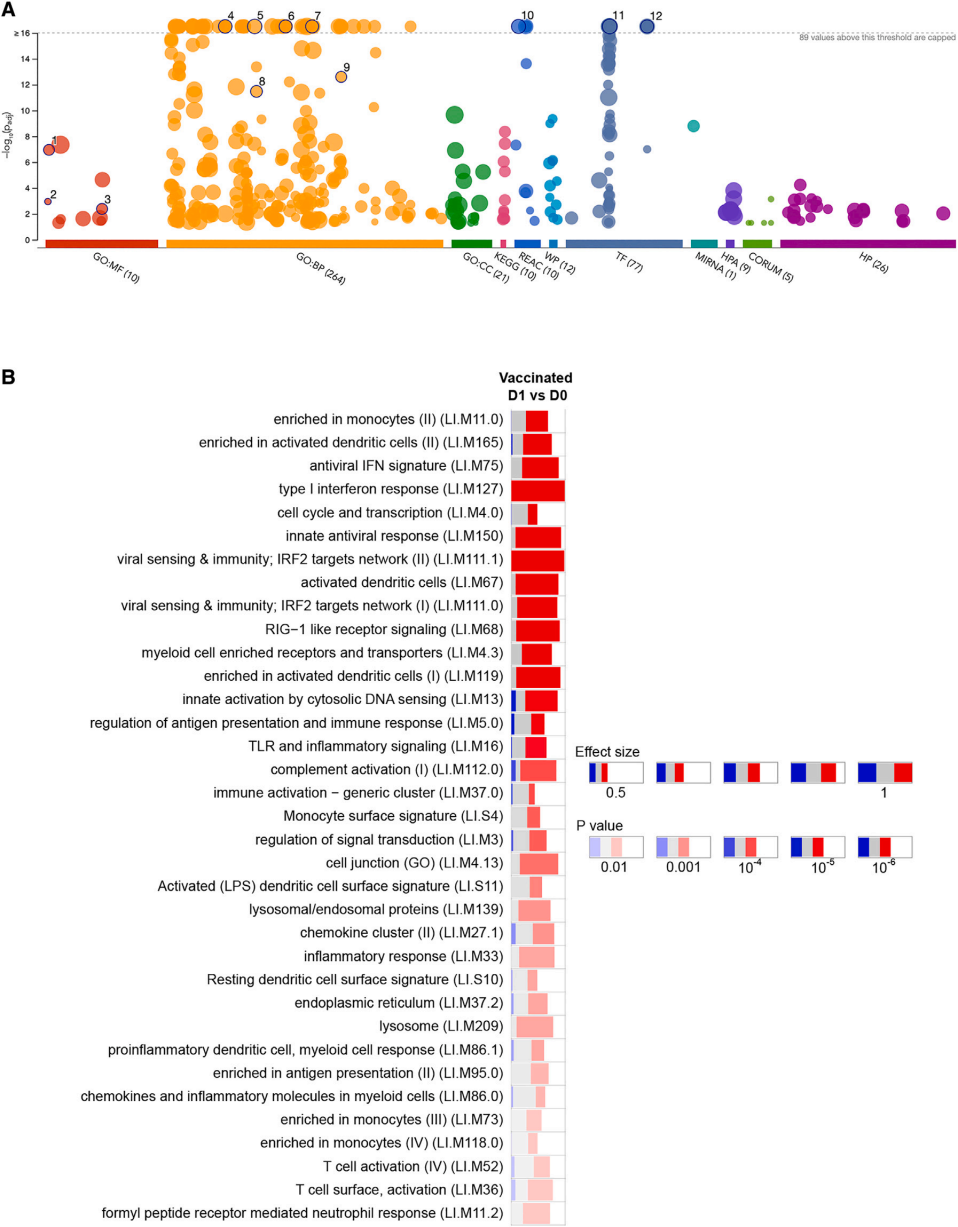
WBTA of vaccine response WBTA was conducted on blood drawn prior to and 1 day post-Vx. Data are shown for 50 vaccinated participants (19 F, 31 M; 9 adult, 41 adolescent). (A) g:Profiler analysis of 311 upregulated genes. Representative enriched pathways are numbered: (1) GO0003725, double-stranded RNA binding; (2) GO0001730, 2′-5′-oligoadenylate synthetase activity; (3) GO0042379, chemokine receptor binding; (4) GO0019221, cytokine-mediated signaling pathway; (5) GO0034097, response to cytokine; (6) GO0045089, positive regulation of innate immune response; (7) GO0051607, defense response to virus; (8) GO0034341, response to type II interferon; (9) GO0071357, cellular response to type I interferon; (10) REAC:R-HAS-1, cytokine signaling in immune system; (11) TF:M00772, IRF motif; and (12) TF:M10080, STAT2 motif. Full details are provided in Table S6. (B) Significantly enriched immune-related modules were identified applying the CERNO test on the adjusted *p* value-ranked lists of genes generated by DeSeq2 (see Table S6 for module gene lists). Bars represent the proportions of significantly upregulated (red), downregulated (blue), or unchanged (gray) genes. The significance of module activation is proportional to the intensity of the bar, while the effect size is proportional to its width. Only the top 35 modules (of 57; see Table S6) are shown for clarity. No DEGs, and hence no modules, were identified in patients receiving placebo.

We used whole-blood transcriptional analysis (WBTA) to confirm vaccine reactogenicity and capacity to induce immune responses in a subset of patients (*n* = 23 placebo and *n* = 27 vaccinated) for whom PAXGene-collected blood samples were available for analysis. Compared to prevaccination, no differentially expressed genes (DEGs) were identified at day 1 post-vaccination in patients receiving placebo (adjusted *p* < 0.05). In contrast, in patients receiving ChAd63-KH, we identified 318 DEGs using this threshold (311 UP, 7 DOWN; Table S6). Using g:Profiler, we identified enriched terms associated with the response to ChAd63-KH, demonstrating activation of antiviral, inflammatory, and immune response genes (Figure 4A; Table S6). To provide a comparison with data from our previous phase 2a study and responses to a range of other vaccines,^33^ we next identified transcriptional modules associated with vaccination. In keeping with the above analysis and our previous analysis of ChAd63-KH responsiveness in patients with PKDL,^31^ highly upregulated transcriptional modules included those associated with interferon responsiveness, dendritic cell and monocyte activation, and antigen presentation (Figure 4B; Table S6). Out of 57 significantly enriched transcriptional modules, 31 were also enriched in the adolescent cohort of the LEISH2a study.^31^ Given that only 2/50 of the patients for whom transcriptomic data were available achieved the primary outcome of 90% clinical response, it was not possible to conduct a robust analysis to identify DEGs or modules associated with clinical cure.

## Discussion

ChAd63-KH is a third-generation vaccine based on adenoviral delivery of a gene construct encoding two well-characterized *Leishmania* antigens. In a phase 2b stand-alone therapeutic trial in Sudanese patients with persistent PKDL, we have confirmed the safety of this vaccine. However, this study failed to demonstrate clinical efficacy when measured by clearance of PKDL lesions over a 90 day follow-up period.

The study was conducted in Gedaref state, South East Sudan, against a backdrop of significant adversity. Over the study period, Sudan sequentially experienced a revolution, a pandemic, a coup, and military conflict that remains ongoing to date. Nevertheless, throughout this period, the trial team was able to maintain most aspects of trial management and governance, although considerable delays in recruitment and the ability to undertake sample analysis occurred. Following consultation with the DSMB and trial sponsor, the decision was taken in November 2022 to curtail recruitment at 86 participants, short of the 100 originally proposed. Full data analysis for the primary clinical outcome is reported here, with additional subgroup analysis being reported where patient metadata or biological samples were incomplete (either due to sample loss or data inaccessibility).

The primary clinical outcome of the study was to determine whether a single administration of ChAd63-KH vaccine was able to induce ≥90% improvement of PKDL in patients with persistent disease of ≥6 month duration. Our analysis of 75 patients that that completed follow-up to day 90 indicated that this was not the case. Similarly, PKDL grade was not improved over this time in vaccinated patients compared to those receiving placebo. Relaxing the criterion for clinical response also did not reveal any significant vaccine effect or effect of prior PKDL duration. Hence, we conclude that this phase 2b clinical trial has not demonstrated efficacy of ChAd63-KH under the conditions tested.

It is therefore important to consider the premise on which this study was based and potential reasons why efficacy was not observed. The immunopathogenic mechanisms that result in the chronic dermal presentation of PKDL are not fully understood. Immunohistological analysis of biopsies from Sudanese patients with PKDL have noted the presence of CD4^+^ and CD8^+^ T cells, but their functional state has not been characterized. Granuloma formation is seen in some cases, but the histopathological picture may not correspond with clinical outcome.^23^ Keratinocyte interleukin (IL)-10 expression during VL was observed to be associated with the later development of PKDL,^34^ but the specific role of IL-10 in PKDL per se is not understood. In Indian PKDL, more extensive studies have characterized CCR4^+^CD8^+^ T cells with a phenotype associated with exhaustion and immune regulation (PD-1^hi^ IL-10^+^).^20^ Collectively, these findings lend support to the hypothesis underpinning this trial, i.e., that enhancing CD8^+^ T cell activation through vaccination may augment the ability of the host to clear parasites/antigens and reduce local pathology. Although we were unable to study antigen-specific T cell responses in this study due to the loss of all frozen cells, our previous studies have indicated that KMP-11- and HASPB-specific CD8^+^ interferon (IFN)γ^+^ responses are induced in patients with PKDL^31^ and healthy volunteers^30^ following ChAd63-KH vaccination. Given the comparability of the transcriptional response to vaccination seen in this and our other studies, we have no reason to suspect that similar T cell responses were not induced in this patient group. However, in the absence of efficacy, it is difficult to ascertain whether this CD8^+^ T cell response was merely insufficient in magnitude to elicit a clinical response or whether a broader response (in terms of antigen specificity or immune response quality) might be needed to achieve a clinical response. Broader antigen exposure along with concurrent inflammation are features of both naturally acquired immunity and that afforded by “leishmanization” and provide a rationale for developing live attenuated vaccines.^35^

With their known ability to rapidly induce antigen-specific CD8^+^ T cells, adenovirus-vectored vaccines have been extensively studied as potential therapeutic vaccines in cancer and chronic hepatitis B virus (HBV) infection. However, studies in these other diseases have, to date, also met with limited success. While a poor choice of immunogens was previously mooted as the explanation for therapeutic failure in cancer therapeutic trials, a common attribute of cancer and chronic viral disease is the development of a microenvironment that favors immune regulation or suppression. These changes often reflect the outcome of signaling through inhibitory checkpoint pathways (e.g., PD-1) or the induction of metabolic checkpoints such as IDO1, and it has been proposed that these disease-associated regulatory constraints on T cell function hinder vaccine efficacy. Experimental evidence to support this notion has been provided by Maini and colleagues, who identified both natural killer cell and PD-1/PD-L1 signaling as negative regulators of HBV vaccine efficacy in animals.^36^ While evidence for such an immunoregulatory environment is less well established in PKDL and other forms of dermal leishmaniasis, some similarities to these other chronic diseases exist. For example, both IDO1 and PD-L1 are abundant in chronic cutaneous leishmaniasis (CL) lesions resulting from *L. donovani* infection in Sri Lanka, with a reduction in PD-L1 expression predicting the rate of cure after antimonial chemotherapy.^37^ More recently, we have identified expression of these checkpoint molecules in CL patients infected with *L.* (*V.*) *braziliensis* and also in patients with PKDL infected with *L. donovani* in India.^38^ PKDL lesions are also enriched in M2 macrophages,^21^ contributing to a lack of leishmanicidal capacity and an immunoregulatory environment. Hence, it is possible that the local inhibitory environment in the skin of patients with persistent PKDL provides an obstacle to effector CD8^+^ T cell function at this site. If this is indeed the case, it is likely that therapeutic vaccination in PKDL might also benefit from interventions that target regulatory pathways, such as those proposed in HBV infection.^36^ While studies using existing biologics or other clinically approved drugs might provide proof-of-concept data in PKDL, it is recognized that such high-cost adjunct therapies are unlikely to drive vaccine implementation in a lower- and middle-income country (LMIC) setting. Alternatively, vaccination in conjunction with antileishmanial chemotherapy should also be considered, given previous successes with immunochemotherapy in PKDL^29^ and more recent evidence suggesting that such chemotherapy may also diminish the immunoregulatory environment in CL patients.^37^

We confirm in this study the potent ability of ChAd63-KH to induce innate responses characterized by antiviral gene signatures and dendritic cell and monocyte activation, with weaker representation of modules associated with neutrophil response and complement activation. The module signatures observed are also in keeping with those observed in a multi-vaccine analysis conducted by Hagan et al.,^33^ which included an adenoviral vaccine candidate (MRKAd5-HIV), and with our previous analysis of patients with PKDL.^31^ In the latter study, we observed that adolescents, but not adults, differentially expressed modules associated with B cell activation (M47.0 and M47.1). However, we did not observe differential expression of these modules in the current study, despite the patient subgroup studied for WBTA being mainly composed of adolescents. The reasons for this difference are currently unknown and subject to ongoing investigation. We had also previously identified 11 whole-blood transcriptional modules predictive of 90% clinical cure, two of which were significantly differentially expressed between cure and non-cure participants (M139: lysosomal/endosomal proteins and M118.0: enriched in monocytes). Unfortunately, as only 2/50 of the samples analyzed here achieved this level of clinical cure, we are unable to confirm or not the predictive value of these modules.

In addition to the loss of samples/data due to the ongoing situation in Sudan and other issues discussed above, a limitation in study design was the evaluation of only one vaccination schedule. ChAd63-KH was administered as a stand-alone therapy using a single dose given intramuscularly. This approach was chosen for both scientific and pragmatic reasons. Extensive data derived from experimental studies and early-phase human trials^39^ and late-phase trials and real-world evidence obtained during the SARS-CoV-2 pandemic^40^ have indicated that repeat dosing with homologous adenovirus vaccines fails to significantly augment the CD8^+^ T cell response. Though direct evidence is lacking, it seems likely that a similar outcome would occur with vaccination in patients with PKDL. Pragmatically, single-dose schedules have multiple benefits in an LMIC setting, including reducing logistical complexities and a reduction in cost, not least due to the savings from not requiring manufacture of two clinical grade vaccines. Similarly, intramuscular dosing is preferred in many clinical situations due to the ease of administration and its standardization and tolerability but may not be the most appropriate route for eliciting skin-homing CD8^+^ T cells.^41^ Thus, we cannot rule out that an efficacy signal may have been observed using a repeat-dosing schedule, by extending the period of follow-up, using a heterologous prime-boost strategy,^39^ and/or varying the route of administration. Given the heterogeneity of clinical presentation and histopathology observed in PKDL, our data also do not rule out the possibility of a therapeutic benefit from ChAd63-KH vaccination in either Sudanese patients with less persistent disease or in patients with PKDL in South Asia. In addition, the prophylactic efficacy of this vaccine against different types of leishmaniasis remains to be evaluated. Such studies would need to be cognizant of the rare AEs reported for Vaxzevria, the ChAdOx1-based SARS-CoV-2 vaccine.^42^ In conclusion, however, this phase 2b study did not provide evidence to support progressing ChAd63-KH as a standalone therapeutic in Sudanese patients with persistent PKDL.

## Materials and methods

### Ethics statement

The LEISH2b study (ClinicalTrials.gov: NCT03969134) was approved by the Sudan National Medicines and Poisons Board and the ethical review committees of the Institute of Endemic Diseases, University of Khartoum, and the Department of Biology, University of York. LEISH2b was sponsored by the University of York. The study was conducted according to the principles of the current revision of the Declaration of Helsinki 2008 and ICH guidelines for GCP (CPMP/ICH/135/95). All participants provided written informed consent before enrollment. Consent forms are available as extended data accompanying the published protocol.^43^

### Study design and participants

LEISH2b was a randomized, double-blind, placebo-controlled therapeutic trial designed to evaluate the safety and efficacy of therapeutic vaccination with the investigational vaccine ChAd63-KH. The initial study design allowed for the recruitment of 100 participants diagnosed with PKDL aged between 18 and 50 (adults) or 12 and 17 (adolescents) and with persistent PKDL of greater than 6 month duration. The full details of the inclusion and exclusion criteria are provided in the published protocol.^43^ Participants were recruited from an endemic area in Gedaref state, Sudan, and all study procedures were conducted at the Professor El-Hassan’s Center for Tropical Medicine, Dooka, Sudan. Clinical monitoring of the study was performed under contract by ClinServ (http://www.clinserv.net). An independent DSMB was established and met throughout the study period. The DSMB charter is available as extended data accompanying the published protocol.^43^

### Eligibility criteria

Full inclusion and exclusion criteria are provided in the protocol.^43^ Key inclusion criteria included the following: age 12–50 years on the day of screening; females must be unmarried, single, or widowed; willing and able to give written informed consent/assent; uncomplicated PKDL of ≥ 6 month duration; otherwise good health; negative for malaria on blood smear; *Leishmania* PCR positive on the screening skin biopsy; and willing to undergo urinary pregnancy tests (females only). Key exclusion criteria included the following: has mucosal or conjunctival PKDL; treatment for PKDL within 21 days; negative rK39 strip test; receipt of a live attenuated vaccine within 60 days or other vaccine within 14 days of screening; history of allergic disease or reactions to vaccines or their components; history of severe local or general reaction to vaccination; fever ≥39.5°C within 48 h; anaphylaxis, bronchospasm, laryngeal edema, collapse, convulsions, or encephalopathy within 48 h; pregnancy, less than 12 weeks post-partum, lactating, or willingness/intention to become pregnant during the study and for 3 months following vaccination (females only); seropositive for hepatitis B surface antigen or hepatitis C (antibodies to HCV); and tuberculosis, leprosy, or malnutrition (malnutrition in adults defined as a BMI < 18.5 and in children and adolescents [8–17 years] as a *Z* score cutoff value of < −2 SD).

### Vaccine and study procedures

As described previously,^31^ ChAd63-KH encodes two leishmanial proteins (KMP-11 and HASPB1) and was manufactured to cGMP by Advent Srl. (Pomezia, Italy; lot B0004; 7.5 × 10^10^ viral particles per mL). ChAd63-KH or placebo (saline) was administered as a single dose in 1 mL volume intramuscularly into the deltoid muscle. Participants were monitored in a hospital for 7 days post-vaccination and thereafter as out patients on days 21, 42, 90, and 120 post-vaccination. Recruitment occurred over four rounds, punctuated by a revolution, a pandemic, and a coup. Meetings of the independent DSMB were held at the end of each round of recruitment. Participants were evaluated for clinical response at days 42 and 90. Those with less than 75% improvement at day 90 were offered standard treatment (AmBisome; 2.5 mg/kg/day for 20 days). If improvement was between 75% and 90%, they were offered conservative treatment or AmBisome, and those with greater than 90% clinical improvement were deemed to not require further treatment (and scored as clinical cure). Some participants defaulted from scheduled visits and were evaluated and treated at unscheduled visits based on their availability. Decisions to treat and evaluation of PKDL were performed by two clinicians. The clinical grade of PKDL was also recorded before and after vaccination using the 4 point grading system described previously.^32^

### Randomization and blinding

A computer-generated (Stata 16) randomization list was prepared by the trial statistician, allocating participants 1:1 to either the active or placebo injection, using randomly permuted blocks, stratified by age group (adult or adolescent). The randomization allocation was communicated to pharmacy staff at the study site through preprepared, sealed envelopes with only external sequential participant numbers. During the trial, adults were found to be more difficult to recruit, and the initial aim of an equal number of adolescents and adults in the trial was not feasible. However, the number of available randomization envelopes in Sudan was limited for each age group. Therefore, once allocations for the adolescent group had been used up, allocations for the adult stratum were used in order of presentation irrespective of age group, thus creating a single, unstratified randomized sample.

The vaccine and placebo injections were prepared in blacked-out syringes labeled only with the participant identification number. The participants and clinical investigators (who administered the IMP and conducted the study follow-up) were blinded as to which injection participants received. The trial statistician (who generated the randomization list), trial coordinator (who prepared sealed envelopes according to the randomization list), assistant pharmacist (who prepared the IMP), and study nurse (who delivered the IMP to the clinical investigators) were not blind to the treatment allocation.

### Outcomes

Primary outcome measures were (1) safety, as recorded as AEs from clinical examination and evaluation of blood biochemistry and hematology, and (2) efficacy, as determined by the proportion of participants reaching a 90% improvement in clinical disease. Secondary outcomes included trajectory of lesion improvement and measures of vaccine-induced immune response. Planned immune analyses included whole-blood transcriptomics and analysis of peripheral lymphocyte responses (antibody and IFNγ ELISpot). Peripheral lymphocyte and antibody responses could not be measured due to sample loss resulting from the military conflict in Sudan.

### Statistical analysis

The statistical analysis plan is provided in the supplemental information. A total sample of size of 100 (randomly assigned 1:1 to be vaccinated with ChAd63-KH or placebo) was determined to be sufficient to detect an increase of ≥25% in the proportion of participants achieving clinical cure, assuming 90% power, 5% statistical significance, a spontaneous clearance rate of ≤2%, and loss to follow-up of ≤5% (Fisher’s exact test). Baseline characteristics were summarized descriptively. Continuous measures are reported as mean, SD, median, and range, while categorical data are reported as counts and percentages. The numbers of local and systemic AEs per participant are presented as median, minimum, maximum, and interquartile range. The median numbers of AEs per participant (separately for local and systemic events) were compared between groups using the Mann-Whitney U test. A primary efficacy outcome of >90% clinical improvement between the two arms was evaluated using a relative RR with statistical significance determined by Fisher’s exact test. Event numbers were too low to allow for an adjusted regression analysis. Comparison of categorical data across groups was analyzed using Fisher’s exact test. Analyses were performed using Stata v.18, R v.4.3.2, or Prism 10 for macOS (v.10.1.1; GraphPad).

### Whole-blood transcriptomic analysis

Whole-blood samples (2.5 mL) were collected into PAXGene tubes immediately prior to vaccination and at 1 day post-vaccination. All reagents and equipment for these analyses were supplied by Thermo Fisher Scientific and processes carried out per the manufacturer’s protocols unless otherwise stated. Total RNA was extracted using the PAXgene Blood RNA kit (PreAnalytiX, QIAGEN). RNA was quantified using the Qubit 2.0 Fluorometer with the RNA HS Assay Kit. ∼50 ng of total RNA was used to construct sequencing libraries with the Ion AmpliSeq Transcriptome Human Gene Expression Kit. Libraries were barcoded, purified with 2.5× Agencourt AMPure XP Magnetic Beads (Beckman Coulter), and then quantified using the Ion Library TaqMan Quantitation Kit on a QuantStudio 5. Libraries were diluted to a concentration of about 50 pMol and pooled in groups of 8 for sequencing on Ion PI Chips. Chips were loaded using the Ion Chef System and the IonPI Hi-Q Chef Kit. Sequencing was performed on an Ion Proton Sequencer using an Ion PI Hi-Q Sequencing 200 Kit.

Differential gene expression analysis was performed using DeSeq2. After count data normalization, differential gene expression analysis was performed using pooled day 0 data from the two study cohorts as the baseline for all contrasts. Enrichment of blood transcription modules at each time point in the different groups was assessed with the tmod R package using as an input the lists of DEGs ranked by the *p* value after multiple test correction, as computed by DeSeq2. Significance of module enrichment was assessed using the CERNO statistical test (a modification of Fisher’s combined probability test) and corrected for multiple testing using the Benjamini-Hochberg correction.

### Role of funders

The funders played no part in study design, data collection, analysis, interpretation, writing, or decision to publish. All authors had full access to study data and final responsibility for the decision to submit for publication.

## Data and code availability

Processed gene expression data are available as supplemental information. Raw gene expression data are available from GEO: GSE266275.

## Supplemental information

Document S1. Tables S2–S5

Data S1. Statistical analysis plan

Table S1. Raw data file for participants indicating PKDL grades, duration, and treatment response

Table S6. Transcriptomic data file including raw counts, DEGs, pathway analysis, and module analysis, related to Figure 4

Document S2. Article plus supplemental information
