# Supplementary material for: A randomized, double-blind phase 2b trial to evaluate efficacy of ChAd63-KH for treatment of post kala-azar dermal leishmaniasis
Source: Mol Ther Methods Clin Dev. 2024 Jul 30;32(3):101310. doi: 10.1016/j.omtm.2024.101310 (PMC11381778; doi:10.1016/j.omtm.2024.101310)
Supplement: Document S1. Tables S2–S5 [file mmc1.pdf]

OMTM, Volume *32*

Supplemental information

A randomized, double-blind phase 2b trial to evaluate efficacy of ChAd63-KH for treatment of post kala-azar dermal leishmaniasis

Brima M. Younis, Rebecca Wiggins, Eltahir A.G. Khalil, Mohamed Osman, Francesco Santoro, Chiara Sonnati, Ada Keding, Maria Novedrati, Giorgio Montesi, Ali Noureldein, Elmukashfi T.A. Elmukashfi, Ala Eldin Mustafa, Mohammed Alamin, Mohammed Saeed, Khalid Salman, Ahmed J. Suliman, Amin E.A. Musa, Alison M. Layton, Charles J.N. Lacey, Paul M. Kaye, and Ahmed M. Musa

Table S1: Raw data file for participants indicating PKDL grades, duration and treatment response.

Provided as Excel file.

Table S2: Incidence of Adverse Events by Body System and Preferred Term

Body System and Preferred Term	Group A			Group B		
	Participants with any event n	Events n	Avg. events per participant (total: 44)	Participants with any event n	Events n	Avg. events per participant (total: 42)
**Overall**	**23**	**43**	**0.98**	**20**	**27**	**0.64**
**Local to injection site**	**13**	**13**	**0.30**	**12**	**12**	**0.29**
Pain/discomfort	7	7	0.16	7	7	0.17
Fluid/blood filled blisters	-	-	-	-	-	-
Soft Swelling	6	6	0.14	5	5	0.12
Hard Swelling	-	-	-	-	-	-
Redness/ discolouration	-	-	-	-	-	-
**Systemic**	**14**	**30**	**0.68**	**12**	**15**	**0.36**
Chills/rigor	2	2	0.05	-	-	-
Malaise/abnormal tiredness	3	3	0.07	-	-	-
General muscle ache	2	2	0.05	-	-	-
Fever	1	1	0.02	-	-	-
Headache	6	6	0.14	3	3	0.07
Nausea	3	3	0.07	-	-	-
Vomiting	1	1	0.02	-	-	-
Anaemia	4	4	0.09	-	-	-
Itching	2	2	0.05	-	-	-
Malaria	6	6	0.14	9	10	0.24
Thrombocytopenia	-	-	-	1	1	0.02
Tinea versicolor	-	-	-	1	1	0.02

Table S3: Severity of Adverse Events by Preferred Term

	Group A			Group B		
**Body System and Preferred Term**	**Grade 1 n**	**Grade 2 n**	**Grade 3 n**	**Grade 1 n**	**Grade 2 n**	**Grade 3 n**
**Overall**	**26**	**17**	**0**	**17**	**10**	**0**
**Local to injection site**	**13**	**0**	**0**	**12**	**0**	**0**
Pain/discomfort	7	0	0	7	0	0
Fluid/blood filled blisters	-	-	-	-	-	-
Soft Swelling	6	0	0	5	0	0
Hard Swelling	-	-	-	-	-	-
Redness/ discolouration	-	-	-	-	-	-
**Systemic**	**13**	**17**	**0**	**5**	**10**	**0**
Chills/rigor	1	1	0	-	-	-
Malaise/abnormal tiredness	2	1	0	-	-	-
General muscle ache	1	1	0	-	-	-
Fever	0	1	0	3	0	0
Headache	1	5	0	-	-	-
Nausea	3	0	0	-	-	-
Vomiting	1	0	0	-	-	-
Anaemia	0	4	0	-	-	-
Itching	1	1	0	-	-	-
Malaria	3	3	0	1	9	0
Thrombocytopenia	-	-	-	1	0	0
Tinea versicolor	-	-	-	0	1	0

Table S4: Non-Serious Adverse Events

Sorted by group and days since vaccination

Participant ID	Age	Gender	Days since vaccination	Description	Type	Relationship to Intervention	Grade	Outcome	Treatment
**Group A**									
A002	18	Female	0	Headache	Systemic	Probably related	Grade 2	Recovered	Paracetamol
A002	18	Female	0	Malaise	Systemic	Probably related	Grade 1	Recovered	No
A002	18	Female	0	Nausea	Systemic	Probably related	Grade 1	Recovered	No
A002	18	Female	0	Pain at injection	Local	Probably related	Grade 1	Recovered	No
A004	18	Male	0	Chills	Systemic	Probably related	Grade 1	Recovered	No
A004	18	Male	0	Pain at injection site	Local	Probably related	Grade 1	Recovered	No
A006	25	Female	0	General muscle pain	Systemic	Probably related	Grade 2	Recovered	Paracetamol
A006	25	Female	0	Nausea	Systemic	Probably related	Grade 1	Recovered	No
A008	37	Male	0	Soft swelling	Local	Probably related	Grade 1	Recovered	No
A014	19	Female	0	Soft swelling	Local	Probably related	Grade 1	Recovered	No
A016	21	Male	0	Pain at injection site	Local	Probably related	Grade 1	Recovered	No
Y013	12	Female	0	Soft swelling at injection site	Local	Probably related	Grade 1	Recovered	No
Y021	12	Male	0	Soft swelling	Local	Probably related	Grade 1	Recovered	No
Y029	14	Male	0	Nausea	Systemic	Probably related	Grade 1	Recovered	No
Y031	14	Female	0	Soft swelling	Local	Probably related	Grade 1	Recovered	No
Y032	12	Male	0	Pain at injection site	Local	Probably related	Grade 1	Recovered	No
Y034	12	Male	0	Pain at injection site	Local	Probably related	Grade 1	Recovered	No
Y045	15	Female	0	Soft swelling	Local	Probably related	Grade 1	Recovered	No
Y050	16	Male	0	Headache	Systemic	Possibly related	Grade 1	Recovered	Paracetamol

Participant ID	Age	Gender	Days since vaccination	Description	Type	Relationship to Intervention	Grade	Outcome	Treatment
A002	18	Female	1	General muscle pain	Systemic	Probably related	Grade 1	Recovered	No
A017	30	Male	1	Pain at injection site	Local	Probably related	Grade 1	Recovered	No
Y008	13	Male	1	Headache	Systemic	Probably related	Grade 2	Recovered	Paracetamol
Y008	13	Male	1	Malaise	Systemic	Probably related	Grade 1	Recovered	No
Y008	13	Male	1	Pain at injection site	Local	Probably related	Grade 1	Recovered	No
Y056	17	Male	1	Chills/rigors	Systemic	Probably related	Grade 2	Recovered	Paracetamol
Y056	17	Male	1	Fever	Systemic	Probably related	Grade 2	Recovered	Paracetamol
Y056	17	Male	1	Headache	Systemic	Probably related	Grade 2	Recovered	Paracetamol
Y056	17	Male	1	Vomiting	Systemic	Probably related	Grade 1	Recovered	No
A002	18	Female	3	Itching all over the body	Systemic	Probably related	Grade 2	Recovered	Chlorphenamine
A004	18	Male	10	Itching all over the body	Systemic	Probably related	Grade 1	Recovered	Chlorphenamine
Y029	14	Male	22	Malaria	Systemic	Unrelated	Grade 2	Recovered	Coartem
Y007	17	Female	96	Malaria	Systemic	Unrelated	Grade 1	Recovered	Coartem
Y063	13	Male	96	Anaemia	Systemic	Unrelated	Grade 2	Recovered	Ferrus Fumarate
Y049	12	Female	113	Anaemia	Systemic	Unrelated	Grade 2	Ongoing	Ferrous + Folic acid
Y049	12	Female	113	Headache	Systemic	Unrelated	Grade 2	Recovered	No
Y049	12	Female	113	Malaise	Systemic	Unrelated	Grade 2	Recovered	No
Y049	12	Female	113	Malaria	Systemic	Unrelated	Grade 2	Recovered	Coartem
Y004	12	Female	131	Malaria	Systemic	Unrelated	Grade 1	Recovered	Coartem
Y057	13	Male	133	Anaemia	Systemic	Unrelated	Grade 2	Ongoing	Folic Acid
Y053	12	Male	135	Malaria	Systemic	Unrelated	Grade 1	Recovered	Coartem
Y045	15	Female	139	Anaemia	Systemic	Unrelated	Grade 2	Ongoing	Ferrous + Folic acid
Y045	15	Female	139	Headache	Systemic	Unrelated	Grade 2	Recovered	No
Y045	15	Female	139	Malaria	Systemic	Unrelated	Grade 2	Recovered	Coartem

Participant ID	Age	Gender	Days since vaccination	Description	Type	Relationship to Intervention	Grade	Outcome	Treatment
**Group B**									
A003	26	Male	0	Headache	Systemic	Probably related	Grade 1	Recovered	No
A005	18	Female	40	Headache	Systemic	Possibly related	Grade 1	Recovered	Paracetamol
A005	18	Female	0	Pain at injection site	Local	Probably related	Grade 1	Recovered	No
A011	28	Male	23	Malaria	Systemic	Unrelated	Grade 2	Recovered	Coartem
A019	18	Male	132	Malaria	Systemic	Unrelated	Grade 2	Recovered	Coartem
A019	18	Male	0	Pain at injection site	Local	Probably related	Grade 1	Recovered	No
Y006	15	Female	96	Malaria	Systemic	Unrelated	Grade 2	Recovered	Coartem
Y010	12	Male	18	Malaria	Systemic	Unlikely to be related	Grade 1	Recovered	Coartem
Y015	12	Female	0	Soft swelling at injection site	Local	Probably related	Grade 1	Recovered	No
Y017	12	Female	0	Soft swelling at injection site	Local	Probably related	Grade 1	Recovered	No
Y022	12	Male	21	Malaria	Systemic	Unrelated	Grade 2	Recovered	Coartem
Y022	12	Male	96	Malaria	Systemic	Unrelated	Grade 2	Recovered	Coartem
Y022	12	Male	0	Soft swelling	Local	Possibly related	Grade 1	Recovered	No
Y023	15	Female	43	Malaria	Systemic	Unrelated	Grade 2	Recovered	Coartem
Y024	15	Female	0	Soft swelling	Local	Probably related	Grade 1	Recovered	No
Y027	15	Male	0	Soft swelling	Local	Probably related	Grade 1	Recovered	No
Y036	12	Female	0	Pain at injection site	Local	Probably related	Grade 1	Recovered	No
Y039	16	Male	0	Pain at injection site	Local	Probably related	Grade 1	Recovered	No
Y041	12	Male	139	Malaria	Systemic	Unrelated	Grade 2	Recovered	Coartem
Y042	12	Male	142	Headache	Systemic	Unrelated	Grade 1	Recovered	No
Y042	12	Male	142	Malaria	Systemic	Unrelated	Grade 2	Recovered	Coartem
Y042	12	Male	0	Pain at injection site	Local	Probably related	Grade 1	Recovered	No
Y047	12	Male	0	Pain at injection site	Local	Probably related	Grade 1	Recovered	No

Participant ID	Age	Gender	Days since vaccination	Description	Type	Relationship to Intervention	Grade	Outcome	Treatment
Y058	12	Male	0	Pain at injection	Local	Probably related	Grade 1	Recovered	No
Y064	14	Male	96	Tinea versicolor	Systemic	Unrelated	Grade 2	Ongoing	Clotrimazole cream
Y065	13	Female	131	Malaria	Systemic	Unrelated	Grade 2	Recovered	Coartem Tabs
Y065	13	Female	131	Thrombocytopenia	Systemic	Unrelated	Grade 1	Ongoing	No

Table S5: Vital Signs and Laboratory Tests Summary

Raw Data

		Group A						Group B					
**Tests**	**Study Visits**	**N**	**Mean**	**SD**	**Min**	**Med**	**Max**	**N**	**Mean**	**SD**	**Min**	**Med**	**Max**
**Vital Signs**													
Blood Pressure Systolic	Screening	44	106.55	7.053	100.0	110.0	120.0	42	105.95	6.270	90.0	110.0	120.0
	Vaccination (before)	44	108.41	8.337	100.0	110.0	130.0	42	107.98	8.700	90.0	110.0	120.0
	Vaccination (2h after)	44	108.18	8.700	90.0	110.0	120.0	42	106.31	6.989	100.0	105.0	120.0
	Visit (D1)	44	108.75	7.860	90.0	110.0	130.0	42	107.98	7.330	100.0	110.0	120.0
	Visit (D3)	44	108.86	7.840	90.0	110.0	120.0	42	107.98	8.268	90.0	110.0	120.0
	Visit (D7)	44	109.32	7.594	90.0	110.0	120.0	42	108.45	7.529	100.0	110.0	120.0
	Visit (D21)	43	107.21	7.966	90.0	110.0	130.0	41	107.32	7.340	100.0	110.0	120.0
	Visit (D42)	42	108.81	6.700	100.0	110.0	120.0	39	106.54	6.992	100.0	110.0	120.0
	Visit (D90)	40	108.75	9.111	100.0	110.0	140.0	35	106.71	7.568	90.0	110.0	120.0
	Visit (D120)	37	106.89	8.110	100.0	105.0	130.0	35	105.57	8.469	90.0	105.0	120.0
Blood Pressure Diastolic	Screening	44	67.73	7.270	60.0	70.0	80.0	42	67.02	6.055	60.0	70.0	80.0
	Vaccination (before)	44	70.11	7.662	55.0	70.0	90.0	42	70.00	6.533	60.0	70.0	80.0
	Vaccination (2h after)	44	68.64	6.413	60.0	70.0	80.0	42	68.10	7.321	55.0	70.0	80.0
	Visit (D1)	44	69.21	7.388	60.0	70.0	80.0	42	67.86	7.897	50.0	70.0	80.0
	Visit (D3)	44	68.43	7.825	55.0	70.0	80.0	42	68.69	6.631	60.0	70.0	80.0
	Visit (D7)	44	69.43	6.923	55.0	70.0	80.0	42	69.76	7.153	60.0	70.0	80.0
	Visit (D21)	43	67.67	7.665	60.0	70.0	80.0	41	69.15	7.323	60.0	70.0	80.0
	Visit (D42)	42	66.88	6.232	60.0	67.0	80.0	39	67.05	6.039	60.0	65.0	80.0
	Visit (D90)	40	67.75	7.067	60.0	67.5	90.0	35	65.71	5.443	60.0	65.0	80.0
	Visit (D120)	37	68.38	6.672	60.0	65.0	80.0	35	68.43	6.391	60.0	70.0	80.0
Temperature	Screening	44	36.46	0.362	35.8	36.4	37.1	42	36.44	0.454	35.6	36.4	38.1
	Vaccination (before)	44	36.33	0.565	35.0	36.4	37.3	42	36.36	0.478	35.4	36.4	37.4
	Vaccination (2h after)	44	36.51	0.389	35.2	36.5	37.1	42	36.51	0.380	35.6	36.5	37.1
	Visit (D1)	44	36.47	0.549	35.4	36.4	38.5	42	36.47	0.442	35.0	36.5	37.1
	Visit (D3)	44	36.52	0.513	35.4	36.6	37.3	42	36.51	0.395	35.5	36.5	37.3

		Group A						Group B					
**Tests**	**Study Visits**	**N**	**Mean**	**SD**	**Min**	**Med**	**Max**	**N**	**Mean**	**SD**	**Min**	**Med**	**Max**
	Visit (D7)	44	36.38	0.559	35.5	36.3	38.8	42	36.40	0.398	35.7	36.4	37.1
	Visit (D21)	43	36.46	0.365	35.7	36.5	37.2	41	36.45	0.447	35.7	36.4	37.4
	Visit (D42)	42	36.39	0.382	35.8	36.4	37.1	39	36.43	0.492	35.3	36.4	37.3
	Visit (D90)	40	36.57	0.417	35.8	36.6	37.7	35	36.51	0.395	35.7	36.5	37.3
	Visit (D120)	37	36.59	0.444	35.8	36.5	37.6	35	36.51	0.558	35.8	36.5	38.5
Pulse	Screening	44	80.11	6.229	68.0	80.0	100.0	42	81.79	5.629	68.0	81.0	92.0
	Vaccination (before)	44	76.71	6.025	65.0	76.0	100.0	42	79.10	7.077	70.0	77.0	98.0
	Vaccination (2h after)	44	78.50	6.245	60.0	79.0	90.0	42	79.86	7.118	60.0	80.0	98.0
	Visit (D1)	44	80.61	7.992	70.0	80.0	100.0	42	80.69	6.323	70.0	81.0	90.0
	Visit (D3)	44	79.30	5.564	70.0	80.0	92.0	42	80.21	6.107	70.0	80.0	92.0
	Visit (D7)	44	79.16	7.278	64.0	80.0	96.0	42	79.74	6.409	70.0	80.0	94.0
	Visit (D21)	43	80.09	5.433	68.0	80.0	90.0	41	78.95	6.008	60.0	80.0	88.0
	Visit (D42)	42	80.12	5.255	68.0	80.0	90.0	39	79.31	4.753	68.0	80.0	88.0
	Visit (D90)	40	80.63	5.977	68.0	80.0	90.0	35	80.80	5.764	68.0	80.0	90.0
	Visit (D120)	37	81.46	5.786	68.0	80.0	92.0	35	80.89	7.190	68.0	80.0	95.0
Respiratory Rate	Screening	44	17.86	2.216	14.0	18.0	24.0	42	18.05	1.899	14.0	18.0	22.0
	Vaccination (before)	44	18.98	2.029	16.0	18.0	24.0	42	18.76	2.184	14.0	18.0	24.0
	Vaccination (2h after)	44	18.91	2.301	12.0	20.0	22.0	42	18.64	1.885	14.0	18.0	24.0
	Visit (D1)	44	18.11	2.071	14.0	18.0	22.0	42	18.02	1.919	14.0	18.0	22.0
	Visit (D3)	44	18.36	2.616	12.0	18.0	25.0	42	18.00	2.306	14.0	18.0	22.0
	Visit (D7)	44	17.34	2.964	12.0	18.0	25.0	42	17.52	2.244	12.0	18.0	22.0
	Visit (D21)	43	17.74	2.205	12.0	18.0	24.0	41	18.02	2.219	12.0	18.0	22.0
	Visit (D42)	42	17.93	2.341	12.0	18.0	22.0	39	17.77	2.400	14.0	18.0	22.0
	Visit (D90)	40	17.28	2.025	14.0	18.0	22.0	35	17.60	2.291	14.0	18.0	22.0
	Visit (D120)	37	17.95	2.321	14.0	18.0	22.0	35	17.80	1.844	14.0	18.0	20.0
**Biochemistry**													
ALT	Screening	44	23.42	13.370	7.0	20.3	77.1	42	25.18	19.175	5.4	20.4	108.0
	Visit (D1)	44	23.51	14.573	6.6	20.4	78.0	42	23.49	16.299	11.6	19.7	93.0
	Visit (D7)	44	22.70	12.009	5.3	18.6	56.5	42	23.31	15.957	11.0	19.8	89.0
	Visit (D90)	40	24.00	12.692	7.0	20.1	66.3	35	21.40	9.321	9.2	19.2	53.0

		Group A						Group B					
**Tests**	**Study Visits**	**N**	**Mean**	**SD**	**Min**	**Med**	**Max**	**N**	**Mean**	**SD**	**Min**	**Med**	**Max**
	Visit (D120)	37	23.66	10.871	7.0	21.1	59.0	35	22.20	8.699	9.4	19.8	48.0
AST	Screening	44	27.88	10.989	10.5	26.8	60.7	42	27.06	13.486	11.4	23.7	71.0
	Visit (D1)	44	27.08	12.922	11.4	24.8	76.9	42	26.35	13.094	13.9	22.9	77.0
	Visit (D7)	44	25.39	8.016	12.5	23.2	49.4	42	25.68	11.925	14.0	23.8	78.0
	Visit (D90)	40	27.72	10.516	12.0	24.1	52.3	35	23.92	6.233	13.0	21.4	43.0
	Visit (D120)	37	28.43	10.745	9.6	28.0	66.0	35	26.52	7.377	13.0	23.4	44.0
Creatinine	Screening	44	0.47	0.203	0.10	0.41	1.10	42	0.42	0.170	0.19	0.40	0.90
	Visit (D1)	44	0.41	0.191	0.20	0.39	1.05	42	0.37	0.143	0.11	0.34	0.75
	Visit (D7)	44	0.39	0.142	0.20	0.35	0.80	42	0.37	0.176	0.14	0.35	0.94
	Visit (D90)	40	0.44	0.217	0.12	0.40	1.20	35	0.39	0.136	0.16	0.35	0.72
	Visit (D120)	37	0.42	0.199	0.18	0.40	1.10	35	0.37	0.142	0.10	0.40	0.73
Albumin	Screening	44	4.31	0.353	3.50	4.30	4.90	42	4.30	0.409	3.60	4.20	5.65
	Visit (D1)	44	4.21	0.333	3.70	4.20	4.90	42	4.17	0.380	3.40	4.15	5.10
	Visit (D7)	44	4.17	0.315	3.40	4.20	5.00	42	4.11	0.304	3.60	4.05	4.80
	Visit (D90)	40	4.23	0.335	3.60	4.20	4.80	35	4.15	0.323	3.60	4.10	4.70
	Visit (D120)	37	4.34	0.530	3.10	4.30	5.40	35	4.19	0.445	3.50	4.20	5.00
Total Bilirubin	Screening	44	0.58	0.270	0.19	0.55	1.60	42	0.60	0.343	0.20	0.52	1.40
	Visit (D1)	44	0.53	0.269	0.20	0.50	1.70	42	0.50	0.277	0.20	0.40	1.28
	Visit (D7)	44	0.54	0.244	0.20	0.48	1.16	42	0.55	0.296	0.20	0.49	1.44
	Visit (D90)	40	0.46	0.159	0.17	0.44	0.83	35	0.46	0.214	0.16	0.40	0.95
	Visit (D120)	37	0.56	0.271	0.20	0.52	1.30	35	0.51	0.291	0.12	0.44	1.20
Direct Bilirubin	Screening	44	0.13	0.117	0.00	0.10	0.60	42	0.17	0.212	0.01	0.10	0.96
	Visit (D1)	44	0.13	0.132	0.00	0.10	0.64	42	0.11	0.131	0.00	0.07	0.47
	Visit (D7)	44	0.10	0.082	0.00	0.09	0.30	42	0.11	0.104	0.00	0.09	0.50
	Visit (D90)	40	0.09	0.061	0.00	0.09	0.20	35	0.11	0.063	0.00	0.10	0.27
	Visit (D120)	37	0.15	0.177	0.00	0.10	0.88	35	0.12	0.124	0.00	0.10	0.60
**Haematology**													
Haemoglobin	Screening	44	13.32	1.233	9.9	13.2	16.8	42	13.11	0.877	11.4	13.1	15.6
	Visit (D1)	44	13.08	1.190	10.0	12.9	16.3	42	12.99	0.896	10.4	13.1	15.5
	Visit (D7)	44	13.22	1.227	9.9	13.1	16.5	42	13.08	0.989	11.2	13.0	16.2

		Group A						Group B					
Tests	Study Visits	N	Mean	SD	Min	Med	Max	N	Mean	SD	Min	Med	Max
	Visit (D90)	40	12.96	1.504	9.3	12.9	16.8	35	12.81	0.997	10.4	13.0	14.7
	Visit (D120)	37	12.60	1.673	8.5	12.4	16.2	35	12.70	1.154	9.5	13.0	14.5
White blood cells	Screening	44	6.02	1.621	3.6	5.9	10.2	42	6.49	1.544	3.3	6.5	11.5
	Visit (D1)	44	5.80	1.151	3.8	5.7	8.8	42	6.04	1.257	3.3	6.1	9.2
	Visit (D7)	44	5.70	1.217	3.3	5.6	9.7	42	6.03	1.517	3.4	5.8	10.5
	Visit (D90)	40	6.21	1.597	3.9	5.9	11.7	35	6.72	1.645	3.6	6.7	10.6
	Visit (D120)	37	5.86	1.416	4.1	5.6	9.6	35	6.00	1.578	3.8	5.8	10.0
Neutrophils	Screening	44	2.62	1.044	1.3	2.4	6.5	42	2.85	0.988	0.9	2.8	5.3
	Visit (D1)	44	3.22	1.039	1.5	3.1	5.9	42	2.77	0.933	1.4	2.7	5.2
	Visit (D7)	44	2.49	0.866	1.4	2.4	5.2	42	2.86	1.163	1.2	2.8	7.5
	Visit (D90)	40	2.79	1.429	1.4	2.5	10.0	35	3.26	1.053	1.2	3.4	6.7
	Visit (D120)	37	2.72	0.843	1.4	2.7	4.8	35	2.95	1.115	1.3	2.8	6.8
Lymphocytes	Screening	44	2.90	1.101	1.5	2.6	7.3	42	2.97	0.800	1.4	2.8	5.4
	Visit (D1)	44	2.01	0.625	0.8	1.9	3.5	42	2.66	0.595	1.3	2.6	3.8
	Visit (D7)	44	2.62	0.779	1.1	2.4	4.7	42	2.51	0.589	1.4	2.5	4.1
	Visit (D90)	40	2.74	0.855	1.3	2.8	5.2	35	2.71	0.901	0.2	2.6	5.0
	Visit (D120)	37	2.62	0.869	1.0	2.4	5.0	35	2.52	0.750	1.3	2.4	4.2
Platelets	Screening	44	296.59	66.361	178.0	296.0	437.0	42	332.24	99.642	158.0	327.0	638.0
	Visit (D1)	44	264.09	68.155	136.0	260.0	414.0	42	322.67	87.629	163.0	309.5	594.0
	Visit (D7)	44	280.14	66.329	153.0	292.0	426.0	42	323.64	85.216	143.0	303.0	491.0
	Visit (D90)	40	294.68	65.658	178.0	293.5	442.0	35	347.63	97.370	139.0	329.0	626.0
	Visit (D120)	37	290.14	77.797	176.0	273.0	470.0	35	320.23	99.209	86.0	309.0	526.0

Change Since Screening

		Group A						Group B					
**Tests**	**Study Visits**	**N**	**Mean**	**SD**	**Min**	**Med**	**Max**	**N**	**Mean**	**SD**	**Min**	**Med**	**Max**
**Vital Signs**													
Blood Pressure Systolic	Screening												
	Vaccination (before)	44	1.86	6.561	-10.0	0.0	20.0	42	2.02	7.656	-10.0	0.0	20.0
	Vaccination (2h after)	44	1.64	6.796	-20.0	0.0	10.0	42	0.36	6.931	-10.0	0.0	20.0
	Visit (D1)	44	2.21	6.604	-10.0	0.0	20.0	42	2.02	6.055	-10.0	0.0	10.0
	Visit (D3)	44	2.32	5.556	-10.0	0.0	20.0	42	2.02	6.055	-10.0	0.0	20.0
	Visit (D7)	44	2.77	5.339	-10.0	0.0	10.0	42	2.50	5.972	-10.0	0.0	10.0
	Visit (D21)	43	0.98	7.042	-18.0	0.0	20.0	41	1.46	5.836	-10.0	0.0	10.0
	Visit (D42)	42	2.43	6.081	-10.0	0.0	10.0	39	0.64	5.640	-10.0	0.0	10.0
	Visit (D90)	40	2.30	6.509	-10.0	0.0	30.0	35	1.29	5.333	-10.0	0.0	10.0
	Visit (D120)	37	0.46	6.834	-10.0	0.0	20.0	35	-0.14	6.472	-20.0	0.0	10.0
Blood Pressure Diastolic	Screening												
	Vaccination (before)	44	2.39	7.662	-10.0	0.0	20.0	42	2.98	6.154	-10.0	0.0	20.0
	Vaccination (2h after)	44	0.91	6.313	-10.0	0.0	20.0	42	1.07	5.795	-10.0	0.0	15.0
	Visit (D1)	44	1.48	7.966	-10.0	0.0	20.0	42	0.83	5.729	-10.0	0.0	10.0
	Visit (D3)	44	0.71	7.593	-10.0	0.0	20.0	42	1.67	5.372	-10.0	0.0	20.0
	Visit (D7)	44	1.71	7.544	-10.0	0.0	20.0	42	2.74	5.968	-10.0	0.0	15.0
	Visit (D21)	43	0.23	7.396	-20.0	0.0	20.0	41	2.07	6.223	-10.0	0.0	10.0
	Visit (D42)	42	-0.50	6.754	-20.0	0.0	20.0	39	0.13	4.658	-10.0	0.0	10.0
	Visit (D90)	40	0.25	6.197	-10.0	0.0	20.0	35	-1.29	4.902	-10.0	0.0	10.0
	Visit (D120)	37	0.54	7.244	-20.0	0.0	20.0	35	1.43	4.300	-10.0	0.0	10.0
Temperature	Screening												
	Vaccination (before)	44	-0.13	0.721	-2.0	0.0	1.1	42	-0.08	0.645	-1.5	-0.1	1.3
	Vaccination (2h after)	44	0.06	0.547	-1.8	0.1	1.1	42	0.07	0.651	-2.5	0.1	1.2
	Visit (D1)	44	0.01	0.617	-1.4	0.1	1.9	42	0.03	0.639	-1.8	0.1	0.9
	Visit (D3)	44	0.06	0.531	-1.1	0.1	1.1	42	0.08	0.551	-1.2	0.2	0.9
	Visit (D7)	44	-0.08	0.531	-1.2	-0.1	1.7	42	-0.04	0.592	-2.1	0.0	1.0
	Visit (D21)	43	0.01	0.491	-0.9	0.0	1.0	41	0.02	0.536	-1.0	-0.1	1.0

		Group A						Group B					
**Tests**	**Study Visits**	**N**	**Mean**	**SD**	**Min**	**Med**	**Max**	**N**	**Mean**	**SD**	**Min**	**Med**	**Max**
	Visit (D42)	42	-0.08	0.545	-1.3	0.0	1.0	39	-0.01	0.661	-1.8	0.0	1.3
	Visit (D90)	40	0.12	0.504	-1.0	0.3	0.9	35	0.06	0.552	-1.7	0.1	0.9
	Visit (D120)	37	0.16	0.508	-0.9	0.2	1.3	35	0.05	0.615	-1.1	0.0	1.9
Pulse	Screening												
	Vaccination (before)	44	-3.41	7.913	-20.0	-2.5	28.0	42	-2.69	7.511	-18.0	-4.0	18.0
	Vaccination (2h after)	44	-1.61	8.911	-20.0	0.0	16.0	42	-1.93	7.906	-20.0	0.0	14.0
	Visit (D1)	44	0.50	9.515	-20.0	0.0	22.0	42	-1.10	7.180	-18.0	0.5	10.0
	Visit (D3)	44	-0.82	7.711	-18.0	0.0	16.0	42	-1.57	7.232	-18.0	0.0	12.0
	Visit (D7)	44	-0.96	8.926	-20.0	0.0	16.0	42	-2.05	6.262	-14.0	-2.0	10.0
	Visit (D21)	43	-0.30	7.704	-24.0	0.0	16.0	41	-2.88	6.218	-14.0	-4.0	8.0
	Visit (D42)	42	0.48	6.545	-14.0	0.0	16.0	39	-2.62	4.832	-13.0	-2.0	8.0
	Visit (D90)	40	0.75	7.232	-18.0	0.0	16.0	35	-1.97	6.392	-20.0	-2.0	12.0
	Visit (D120)	37	0.57	6.890	-20.0	0.0	20.0	35	-1.77	6.722	-18.0	0.0	10.0
Respiratory Rate	Screening												
	Vaccination (before)	44	1.11	1.907	-2.0	2.0	6.0	42	0.71	2.266	-2.0	0.0	9.0
	Vaccination (2h after)	44	1.05	2.362	-4.0	1.5	6.0	42	0.60	2.275	-4.0	0.0	6.0
	Visit (D1)	44	0.25	2.686	-8.0	0.0	6.0	42	-0.02	2.414	-6.0	0.0	4.0
	Visit (D3)	44	0.50	2.445	-8.0	0.0	7.0	42	-0.05	2.603	-6.0	0.0	6.0
	Visit (D7)	44	-0.52	3.038	-10.0	0.0	7.0	42	-0.52	2.422	-6.0	0.0	6.0
	Visit (D21)	43	-0.16	2.400	-8.0	0.0	6.0	41	-0.02	2.329	-5.0	0.0	4.0
	Visit (D42)	42	-0.02	2.884	-6.0	0.0	4.0	39	-0.44	2.303	-4.0	0.0	4.0
	Visit (D90)	40	-0.63	2.382	-6.0	0.0	4.0	35	-0.51	2.582	-6.0	0.0	4.0
	Visit (D120)	37	0.05	2.758	-6.0	0.0	4.0	35	-0.26	2.077	-6.0	0.0	4.0
**Biochemistry**													
ALT	Screening												
	Visit (D1)	44	0.08	6.812	-23.4	-0.5	29.1	42	-1.69	6.291	-28.9	-0.7	11.0
	Visit (D7)	44	-0.73	6.215	-20.6	-1.0	15.9	42	-1.87	8.162	-28.7	-1.9	24.6
	Visit (D90)	40	0.47	11.291	-30.2	0.2	42.2	35	-5.00	15.054	-55.0	-1.8	11.8
	Visit (D120)	37	-0.34	11.872	-41.1	0.0	39.0	35	-4.29	15.885	-60.0	-0.9	17.2
AST	Screening												

		Group A						Group B					
**Tests**	**Study Visits**	**N**	**Mean**	**SD**	**Min**	**Med**	**Max**	**N**	**Mean**	**SD**	**Min**	**Med**	**Max**
	Visit (D1)	44	-0.80	9.688	-41.4	-1.2	20.6	42	-0.71	7.352	-30.7	-0.1	15.1
	Visit (D7)	44	-2.49	8.277	-39.1	-0.7	10.8	42	-1.39	6.415	-27.7	-0.2	8.0
	Visit (D90)	40	-0.34	10.567	-45.4	0.8	22.6	35	-4.24	11.310	-33.1	-1.5	13.6
	Visit (D120)	37	0.04	12.288	-31.2	1.0	35.0	35	-1.70	11.939	-34.0	0.6	18.0
Creatinine	Screening												
	Visit (D1)	44	-0.06	0.098	-0.23	-0.05	0.14	42	-0.05	0.118	-0.30	-0.06	0.30
	Visit (D7)	44	-0.08	0.168	-0.57	-0.06	0.22	42	-0.05	0.139	-0.35	-0.05	0.26
	Visit (D90)	40	-0.03	0.111	-0.24	-0.04	0.24	35	-0.02	0.112	-0.37	0.00	0.20
	Visit (D120)	37	-0.06	0.142	-0.44	-0.02	0.20	35	-0.04	0.146	-0.30	-0.07	0.23
Albumin	Screening												
	Visit (D1)	44	-0.10	0.272	-0.70	-0.10	0.80	42	-0.13	0.318	-1.35	-0.10	0.80
	Visit (D7)	44	-0.14	0.384	-0.80	-0.10	0.70	42	-0.19	0.331	-1.25	-0.15	0.40
	Visit (D90)	40	-0.10	0.385	-1.00	-0.10	0.50	35	-0.16	0.530	-1.95	0.00	0.70
	Visit (D120)	37	0.05	0.554	-1.40	0.10	0.80	35	-0.10	0.729	-1.75	0.10	0.90
Total Bilirubin	Screening												
	Visit (D1)	44	-0.05	0.216	-0.70	-0.04	0.55	42	-0.10	0.199	-0.90	-0.04	0.20
	Visit (D7)	44	-0.04	0.213	-0.58	-0.01	0.52	42	-0.05	0.294	-0.90	-0.01	1.12
	Visit (D90)	40	-0.11	0.187	-0.77	-0.11	0.25	35	-0.10	0.276	-0.90	-0.07	0.55
	Visit (D120)	37	-0.02	0.221	-0.80	0.00	0.45	35	-0.05	0.275	-0.79	0.01	0.42
Direct Bilirubin	Screening												
	Visit (D1)	44	0.00	0.148	-0.40	0.00	0.57	42	-0.06	0.213	-0.78	0.00	0.28
	Visit (D7)	44	-0.02	0.102	-0.30	0.00	0.15	42	-0.07	0.229	-0.90	0.00	0.25
	Visit (D90)	40	-0.03	0.133	-0.45	-0.02	0.17	35	-0.06	0.223	-0.91	0.00	0.16
	Visit (D120)	37	0.03	0.193	-0.41	0.02	0.79	35	-0.06	0.243	-0.76	0.00	0.58
**Haematology**													
Haemoglobin	Screening												
	Visit (D1)	44	-0.24	0.548	-1.3	-0.3	0.7	42	-0.13	0.403	-1.0	-0.1	0.7
	Visit (D7)	44	-0.10	0.590	-1.4	0.0	1.0	42	-0.03	0.648	-1.4	-0.1	1.7
	Visit (D90)	40	-0.32	0.768	-3.2	-0.4	1.6	35	-0.23	0.880	-2.8	-0.1	1.3
	Visit (D120)	37	-0.70	1.022	-3.5	-0.6	1.2	35	-0.35	0.960	-2.8	-0.3	1.7

		Group A						Group B					
**Tests**	**Study Visits**	**N**	**Mean**	**SD**	**Min**	**Med**	**Max**	**N**	**Mean**	**SD**	**Min**	**Med**	**Max**
White blood cells	Screening												
	Visit (D1)	44	-0.22	1.096	-2.4	-0.1	1.9	42	-0.45	1.157	-3.8	-0.3	1.4
	Visit (D7)	44	-0.32	1.050	-3.1	-0.4	2.2	42	-0.46	1.343	-4.2	-0.5	2.9
	Visit (D90)	40	0.13	1.971	-4.6	0.1	7.5	35	0.09	1.781	-3.6	0.2	4.6
	Visit (D120)	37	-0.23	1.387	-2.6	-0.4	2.7	35	-0.55	1.467	-3.9	-0.6	2.9
Neutrophils	Screening												
	Visit (D1)	44	0.61	0.951	-1.5	0.5	3.6	42	-0.08	0.818	-2.5	0.1	1.7
	Visit (D7)	44	-0.13	0.902	-3.1	-0.1	2.3	42	0.01	1.057	-2.3	0.0	2.8
	Visit (D90)	40	0.14	1.744	-3.5	0.1	7.7	35	0.34	1.169	-1.4	0.1	3.2
	Visit (D120)	37	0.09	1.036	-1.9	0.1	2.1	35	0.10	1.083	-2.2	0.1	2.8
Lymphocytes	Screening												
	Visit (D1)	44	-0.89	0.997	-5.4	-0.7	1.2	42	-0.31	0.643	-2.3	-0.2	0.7
	Visit (D7)	44	-0.28	0.871	-4.2	-0.1	1.4	42	-0.46	0.695	-2.9	-0.5	0.9
	Visit (D90)	40	-0.09	0.658	-1.7	0.0	1.3	35	-0.36	0.949	-2.8	-0.3	1.4
	Visit (D120)	37	-0.34	1.040	-5.0	-0.3	1.6	35	-0.54	0.692	-2.2	-0.5	1.1
Platelets	Screening												
	Visit (D1)	44	-32.5	29.607	-81	-34.5	87.0	42	-9.6	45.649	-192	-11.5	112.0
	Visit (D7)	44	-16.5	33.533	-104	-17.0	64.0	42	-8.6	44.338	-147	-12.5	111.0
	Visit (D90)	40	-6.0	43.646	-127	-2.0	74.0	35	11.4	82.137	-346	22.0	210.0
	Visit (D120)	37	-6.9	51.279	-190	-10.0	87.0	35	-16.7	66.440	-238	-3.0	72.0

Table S6: Transcriptomic data file including raw counts, DEGs, pathway analysis and module analysis (supports Figure 4).

Provided as Excel file.
